# The Effect of Physical Exercise on Cognitive Impairment in Neurodegenerative Disease: From Pathophysiology to Clinical and Rehabilitative Aspects

**DOI:** 10.3390/ijms222111632

**Published:** 2021-10-27

**Authors:** Giacomo Farì, Paola Lunetti, Giovanni Pignatelli, Maria Vittoria Raele, Alessandra Cera, Giulia Mintrone, Maurizio Ranieri, Marisa Megna, Loredana Capobianco

**Affiliations:** 1 Department of Basic Medical Sciences, Neurosciences and Sense Organs, Aldo Moro University, Piazza Umberto I, 70121 Bari, Italy; dr.giacomofari@gmail.com (G.F.); giovannipignatelli88@gmail.com (G.P.); maryvi.92@hotmail.it (M.V.R.); alessandracera@libero.it (A.C.); giuliamintrone@gmail.com (G.M.); maurizio.ranieri@uniba.it (M.R.); marisa.megna@uniba.it (M.M.); 2 Department of Biological and Environmental Science and Technologies (Di.S.Te.B.A.), University of Salento, Piazza Tancredi 7, 73100 Lecce, Italy; paola.lunetti@unisalento.it

**Keywords:** physical activity, exercise, rehabilitation, Alzheimer’s disease, Parkinson’s disease

## Abstract

Neurodegenerative diseases are a group of pathologies that cause severe disability due to motor and cognitive limitations. In particular, cognitive impairment is a growing health and socioeconomic problem which is still difficult to deal with today. As there are no pharmacologically effective treatments for cognitive deficits, scientific interest is growing regarding the possible impacts of healthy lifestyles on them. In this context, physical activity is gaining more and more evidence as a primary prevention intervention, a nonpharmacological therapy and a rehabilitation tool for improving cognitive functions in neurodegenerative diseases. In this descriptive overview we highlight the neurobiological effects of physical exercise, which is able to promote neuroplasticity and neuroprotection by acting at the cytokine and hormonal level, and the consequent positive clinical effects on patients suffering from cognitive impairment.

## 1. Introduction

Neurodegenerative diseases are a group of hereditary disorders of the central nervous system, which cause slow, progressive damage of specific neuron populations and their connections [1]. They can cause severe clinical consequences, such as motor and cognitive disability, and care dependency [2]. 

In particular, the impairment of cognitive abilities is a growing health and socioeconomic problem. In fact, dementia and mild cognitive impairment (MCI) cases are estimated to increase in Europe from 7.7 million in 2001 to 15.9 million in 2040 [3]. According to the estimates provided by the Global Burden of Disease in 2003, dementia contributed to 11.2% of years lived with disability in people aged 60 and over, more than strokes, musculoskeletal disorders, cardiovascular disease and all forms of cancer [4]. Costs of dementia are forecasted to raise in the whole European continent by about 43% between 2008 and 2030, to over EUR 250 billion. Therefore, the research regarding this disease represents a real challenge for physicians and rehabilitators. 

Many treatment strategies have been tested, using both drugs and rehabilitation techniques [5]. Furthermore, great interest has been placed on the importance of lifestyles both in the management and in the prevention of cognitive limitations deriving from neurodegenerative diseases [6]. 

Among the life habits, the possible role of physical activity seems increasingly interesting. There has long been discussion regarding the positive effects of physical exercise (PE) on brain activity [7]. Raichlen et al. reported a positive correlation between the size of the human brain and endurance-exercise capacity, suggesting a coevolution between locomotion and cognition in human [8]. 

However, PE has only recently begun to receive the attention of the international scientific community, especially around its possible effects on cognitive functions, spatial learning and memory, as a nondrug method of maintaining brain health and treating neurodegenerative and psychiatric conditions [9]. The beneficial effects of aerobic and resistance exercises in adult and geriatric populations have been demonstrated [10], and in the same way, rehabilitative therapeutic exercise seems to be an effective instrument to try to slow down the unavoidable progression of cognitive impairment in pathologies such as dementia [11]. So, PE provides a nonpharmacological approach to slowing age-related decline and reducing disease-related cognitive impairment in older adults through the reduction of risk factors and its neuroprotective capacity. Nevertheless, the biochemical and molecular bases underlying the neuroplasticity mechanisms are still partly unclear, as are the processes that translate the effects of PE into neurological and clinical benefits [12].

The aim of this literature descriptive overview is to investigate whether PE has a clinically positive and rehabilitation-improving effect on cognitive impairment related to neurodegenerative diseases, and to understand more deeply the neurobiological mechanisms that give an explanation of these processes.

## 2. Physical Exercise-Related Neurobiological Processes in Neurodegenerative Diseases

**Figure 1:**
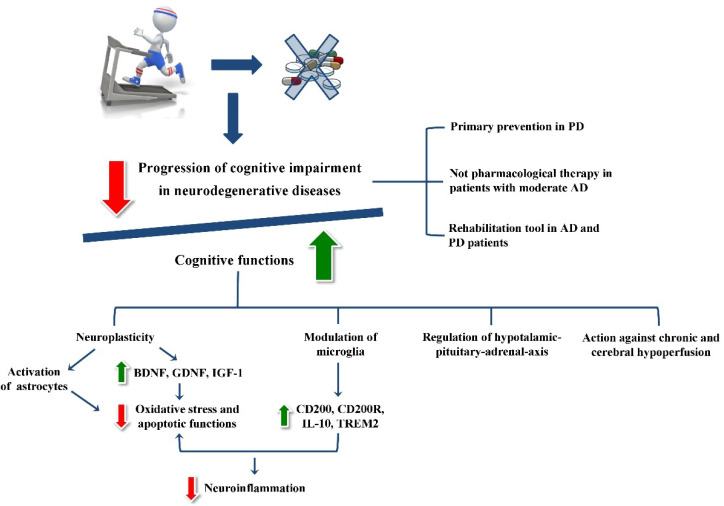
A graphic scheme of all the clinical and neurobiological effects of physical exercise.

The maintenance of cognitive function lies in the processes of neuroplasticity and neuroprotection (Figure 1).

Neuroplasticity is the ability of the brain to alter functional and structural properties in order to respond to changing demands, and it results in learning and acquiring skills [13]. It is well known that PE facilitates neuroplasticity of certain brain regions, and as a result, it improves cognitive functions [14]. Farmer et al. demonstrated that hippocampal neurons grow and develop from a single population of stem cells in response to exercise [15]. New neurons are more flexible in making connections than mature ones, allowing for healthy learning, a good memory and mood regulation [16]. Moreover, to make many brain activities work properly, the hippocampus plays a role through the secretion of some stimulating factors such as brain-derived neurotrophic factor (BDNF), glial-derived neurotrophic factor (GDNF) and insulin-like growth factor (IGF-1), that together with synapsins and synaptophysins induce a downregulation of oxidative stress and apoptotic functions [17]. Increased release of protective neurotrophins is associated with PE in animal and human studies [18].

### 2.1. Neurotrophin Modulation Induced by Physical Exercise

BDNF belongs to a family of small, secreted proteins that also include nerve growth factor, neurotrophin 3 and neurotrophin 4. It acts as an antiapoptotic and antioxidant agent, and tends to suppress all the autophagy processes induced by microglia and proinflammatory cytokines [19]. BDNF stands out among all neurotrophins due to its high expression levels in the brain and its potent effects at synapses [20]. Furthermore, Churchill et al. in 2002 indicated that BDNF neurotrophin is involved in information storage processes in long-term memory and learning [21].

Physiologically, after an ischemic insult, the microglia are activated and acts with proinflammatory activity, leading to an increase in free oxidative radicals (ROS) to which neuronal cell try to respond by consuming ATP and inevitably dying; therefore, increasing the levels of BDNF through PE could reduce the impact of these events [21]. In fact, PE increases the expression of BDNF but also of IGF-1, which interacts with BDNF to mediate exercise-induced cognitive gains [22,23,24]. Mattson et al. suggested that, since endurance exercise clearly increases BDNF expression in the brain, the improvement in exercise capacity may positively enforce brain growth, especially in hippocampus [25]. So, exercise training is known to enhance the neuronal functions of the amygdala and hippocampus, since it seems to increase the levels of BDNF/TrkB signaling molecules [26]. Fahimi et al. reported that around four weeks of treadmill and running wheel exercises in mice brought about many changes such as: significant increases in BDNF-mRNA and protein levels, significantly increased synaptic load in dentate gyrus, changes in the morphology of astrocytes and orientation of astrocytic projections toward dentate gyrus cells [27]. Zsuga et al. suggested also that BDNF modulates neuronal dopamine content and its release, which are essential for neuronal plasticity, neuronal survival, learning and memory [28]. So, BDNF concentration’s increase in blood after PE seems to be a preventive factor with regard to cognitive impairment.

Like BDNF, IGF-1 also promotes neuronal growth, survival and differentiation. Its blood concentration seems to increase in older adults after 6 months of moderate-to-high levels of resistance exercise [29]. Although the specific molecular actions of IGF-1 that contribute to improved cognitive performance in aged animals remain unknown, there is new evidence that synaptic morphology and function is regulated by IGF-1. In fact, Shi et al. quantified total synaptic profiles as well as synaptic profiles in multiple spine bouton (MSB) complexes in the CA1 region of the hippocampus and determined the postsynaptic density (PSD) length. The results indicated a decrease in total synapses between middle and old age, but IGF-1 infusion to old animals increased PSD length and the number of MSB synapses. These changes appear to be morphological correlates of increased synaptic efficacy and suggest that IGF-1 levels influence synaptic function in the CA1 region of the hippocampus [30]. These findings could indicate that circulating IGF-1 is essential for protecting the normal brain function. Moreover, Trejo et al. demonstrated that behavioral and synaptic deficits were improved in IGF-1-deficient mice by prolonged systemic administration of IGF-1, which normalized the density of glutamatergic buttons in the hippocampus. These results indicate that circulating IGF-1 also influences mature brain function, i.e., learning and synaptic plasticity, through its trophic effects on central glutamate synapses [31]. Therefore, raising the levels of circulating IGF-1 through PE is important not only to prevent the appearance of cognitive impairment, but also to improve cognitive performance in those affected.

Recent evidence has highlighted the importance of PE through the action of another molecule, a hormone called irisin. It was identified in 2012 in a study by Bostrom et al. [32]; irisin is generated in skeletal muscle precisely in response to exercise. The main effect of this hormone is to control bone mass, with positive effects on cortical mineral density and geometry that might help to treat osteoporosis. Nevertheless, it seems to produce positive effects on brain function. Although the mechanism of action is not still well known, in rats it has been shown that PE leads to an increase in fibronectin type III domain-containing protein 5 (FNDC5), a membrane protein that, once cleaved, forms the hormone irisin. Once secreted into the extracellular matrix, this hormone binds to its receptor and activates a signaling cascade that induces the expression of the BDNF gene, thus leading to neuroprotection in an indirect manner. As mentioned above, increased BDNF levels improve the health and function of the hippocampus [33]. On the other hand, at the peripheral level, overexpression of FNDC5/irisin recovers memory impairment induced by most neurodegenerative diseases, whereas its blockade at the central or peripheral level attenuates synaptic plasticity and worsens memory in AD mice [34]. Li et al. demonstrated that intravenous injection of irisin reduces levels of active microglia and TNF-α expression, thereby protecting neurons from inflammation [35]. Moreover, the novel exercise-induced hormone seems to protect against neuronal injury through activation of the Ak strain-transforming (AKT) and extracellular signal-regulated kinase 1/2 (ERK1/2) signaling pathways [36]. These results suggest that irisin contributes to the neuroprotective effects of exercise in cerebral ischemia and is a promising agent for the prevention and treatment of ischemic stroke and neurodegenerative diseases.

### 2.2. Impact of Physical Exercise on Astrocytic Functions

Other important factors involved in the neurobiological processes induced by PE are astrocytes, which are the cells most represented in the central nervous system (CNS), belonging to the glia. They modulate the transmission of neuronal signals and integrate information from synapses [37]. Astrocytes have also different functions. Firstly, they are responsible for maintaining brain homeostasis, stabilizing the extracellular concentrations of potassium, chlorine and calcium ions [38]. It is also known that astrocytes can perform protective functions in the CNS by taking excite-toxic glutamate and producing glutathione against oxidative stress, degrading amyloid peptides and regulating cell volume and ionic homeostasis so as to facilitate the repair of the blood–brain barrier (BBB) and regulate inflammation of the CNS [39]. In relation to all these functions, it is understood how possible inefficiencies of these cells can contribute to the pathogenesis of numerous brain disorders such as cognitive impairment. A growing branch of neuroscience has shown that glia directly affects the ability to generate neuronal signals, locally and globally modulating the activity of the brain network [40].

In elderly neurodegenerative disease, such as dementia, the decline of cognitive functions often is a result of alterations in brain circulation [41]. Therefore, PE seems to be able to contrast the damage related to cerebral hypoperfusion. Leardini-Tristao et al. demonstrated how early moderate exercise in chronic hypoperfusion can modulate neuroinflammation, cerebral circulation, and astrocyte coverage. After 12 weeks, early moderate exercise reduces blood pressure and microglial activation in the hippocampus and improves astrocyte coverage in blood vessels of the cerebral cortex [42]. This suggests that early and long-term moderate exercise may represent a nonpharmacological approach in dementia due to chronic hypoperfusion.

### 2.3. The Physical Exercise Modulation of Microglia

The role of PE in neurodegenerative diseases is also carried out through the modulation of microglia, reducing neuroinflammation. Microglia cells are the “resident” macrophages, and are the first line of defense against damage in the CNS. In fact, microglia guarantee the trophism of the neuron, control neuronal plasticity and participate in the control of the BBB, participating also to neuroinflammatory processes [43]. After an ischemic brain damage, microglia rapidly migrate to the injury site and contribute to the inflammatory mechanism by promoting excessive production of inflammatory cytokines and cytotoxic substances. In the absence of external stimuli, the microglia are in an “inactive” state in which; thanks to its branched cell morphology, they constantly monitor the neuronal microenvironment. Once activated, however, they undergo a morphological modification that leads them to assume a mobile amoeboid form in order to reach the place of the insult. The functional phenotypes associated with these two phases are called M1 and M2, and are correlated, respectively, with neurotoxic and neuroprotective functions. Thus, M2 microglia exert a protective role after ischemia through releasing neurotrophic factors including BDNF, IGF-1, interleukin-4 (IL-4), and interleukin-10 (IL-10). M2 microglia can maintain BBB integrity, promote the proliferation and differentiation of neural stem cells (NSCs) and oligodendrocyte progenitor cells (OPCs), and facilitate myelin regeneration and tissue repair. Conversely, M1 microglia can remain active for a long time, releasing cytokines and neurotoxic factors which can in turn contribute to increase the neuronal damage [44]. There is a possible correlation between glial activation, neurodegeneration and dementia. Laakso et al. verified that microglial activation is associated with neuronal damage by demonstrating hippocampal atrophy in patients with chronic neurodegenerative diseases such as AD and Parkinson Disease (PD). Therefore, modulation of neuroinflammation could have important therapeutic implications in these pathologies [45]. The therapeutic role of PE as a regulator of neuroinflammation fits into this context. Indeed, PE induces the production of a series of anti-inflammatory molecules [46]: it increase the expression of Cluster of Differentiation 200 (CD200), an immunomodulatory factor that inhibits microglia by interacting with its receptor CD200R on microglial membranes; moreover, long-term exercise is able to regulate the expression of IL-10, an anti-inflammatory myokine, and increase the levels of soluble triggering receptor expressed on myeloid cells 2 (TREM2), an immunoglobulin receptor that regulates phagocytosis and cytoskeleton rearrangement and has a protective action in the cerebrospinal fluid (CSF) of patients affected by AD. Finally, PE increases antioxidant levels, and this contrasts neuroinflammation caused by microglia [47].

### 2.4. The Influence of Physical Exercise on Hormonal Activity

In recent years, it has also been demonstrated that PE slows the development of neurodegenerative diseases from a hormonal point of view.

One of the main functions of hippocampus consists of the inhibition and adaptive control of hypothalamic–pituitary–adrenal axis (HPA) as a stress response [48]. Physiologically, the hippocampus contains many steroid receptors, divided into mineralocorticoids receptors (MR) and glucocorticoids ones (GR) [49]. It has been demonstrated that a physiological reduction in the number of steroid receptors in different areas of the hippocampus occurs with age [50] and that the HPA tends to free itself from the inhibitory control exerted by the higher centers. Holsboer et al. developed a high-sensitivity test based on the combination of dexamethasone-suppression/corticotropin-releasing hormone stimulation (DEX/CRH test) to study the function of the HPA system. The test results showed that basal cortisol concentration was significantly higher in AD patients than in healthy subjects, and that the minimum drug concentration to which the patient reacts to cortisol was significantly higher in healthy subjects than in the AD patient group. In addition, AD patients released significantly less adrenocorticotropic hormone (ACTH) and cortisol after further CRH stimulation than the control group. These results confirm that the regulation of the HPA system is impaired in AD. The impairment of the HPA axis and the corresponding increase in basal cortisol may be attributed to advancing age and the process of hippocampal destruction, which is typical of neurodegenerative diseases [51]. 

Lanfranco et al. demonstrated that PE also represents a powerful physiological stimulus on the HPA axis [52]. To understand the brain activation in response to PE, a comprehensive analysis was performed on rats after 90 min of treadmill running [53]. The results demonstrated a hypersecretion of CRH, arginine-vasopressin hormone and ACTH, which conduce to an increase in basal adrenal cortisol secretion. Two major factors modulate the HPA axis response to resistance exercise: intensity and duration [54]. The minimum exercise intensity required to produce a cortisol response from HPA axis is 60% of maximum oxygen consumption (VO_2_max); for exercise above 60% VO_2_max, plasma cortisol concentrations increase linearly with exercise intensity [55]. Below this intensity threshold (<60% VO_2_max), ACTH and cortisol concentrations may increase only if 90 min exercise with at least 40% VO_2_max is maintained [56]. When PE is performed, a high response of hormones such as ACTH and cortisol occurs to mitigate the enormous metabolic demand essential to the body to complete the exercise itself [57]. Indeed, cortisol remodels muscle fibers by inhibiting the synthesis of new proteins and stimulating the degradation of the proteins through the ubiquitin pathway; moreover, cortisol affects neuromuscular function through various rapid and short-term mechanisms, such as the regulation of Ca^2+^ channels [58]. Moreover, Klaperski et al. showed that continuous, intense PE led to reduced stress reactivity and improved recovery from neuropsychological stress compared with physically inactive subjects [59]. Finally, adaptation to exercise induced a decreased peripheral tissue sensitivity to GCs that is supposed to protect the body from the severe metabolic and immune consequences of increased cortisol levels [60].

In conclusion, PE is able to determine transcriptional and translational changes at the cellular level, and it also induces a tissue biological stimulus at the CSN level and regulates the hormonal axes involving the brain. It therefore produces a cascade of positive biological and metabolic effects, both in terms of neuroplasticity and in terms of neuroprotection; for these reasons it can be considered a real drug, devoid of any side effects for the CSN.

## 3. Physical Exercise-Related Clinical and Rehabilitative Effects in Neurodegenerative Diseases

At this point it becomes essential to understand how the neurobiological effects of PE translate to a clinical level. Translating the perspective from the cell to the body and its function, from the particular to the general, is the indispensable premise for evaluating the effectiveness of PE as an opportunity for treatment and rehabilitation in the management of the most important neurodegenerative diseases that cause cognitive impairment (Figure 1).

### 3.1. The Role of Physical Exercise in Alzheimer’s Disease (AD)

AD is certainly the most widespread and disabling neurodegenerative disease. AD is a neurodegenerative disease characterized by neuronal and synaptic changes in the cerebral cortex and in some subcortical regions, which cause the deterioration of cognitive and psychobehavioral functions [61]. The epidemiological data available over the last 10 years have shown that PE can slow down the progression of neurodegenerative diseases [62]. Aerobic PE increases cardiac output, and consequently, cerebral blood flow. This mechanism also involves an increase in angiogenesis, neurogenesis, synaptogenesis and the synthesis of neurotransmitters, which in turn improve memory and cognitive functions [63]. Over the years, the correlation between aerobic PE and the improvement of cognitive function in subjects with AD has gained more and more evidence [64,65]. The effects of moderate physical activity on higher brain functions were first highlighted by observing how simple walking, if carried out regularly, leads AD patients to improve their cognitive abilities, a result which is quantifiable by using the Mini Mental State Examination (MMSE) [66]. Furthermore, according to a very large sample study conducted by Norton et al. [67], a percentage equal to 12.7% of AD cases worldwide and 20.3% of AD cases in Europe in 2010 were attributed to physical inactivity. Additionally, the findings of a previous study published by Larson et al. were in line with these data: an analysis was conducted on a sample of 1740 individuals over the age of 65; all were subjected to a regular session of PE carried out for 2 years (15 min/session of walking, cycling, swimming, aerobics, rhythmic gymnastics, water aerobics, strength training, stretching or other activities). For those who exercised three or more times per week, the incidence of dementia was found to be 13.0 per 1000 person-years; on the other hand, for those who exercised less than three times a week, the same incidence rises to 19.7 per 1000 person years. In fact, as reported above, PE improves the cerebral vascular reserve and neuronal plasticity; in a study published by Larson et al. it was demonstrated that forty minutes of PE (ergocycle, treadmill and stair-climbing) four times a week for a period of 12 weeks could increase the cerebral blood flow in the hippocampal dentate gyrus, which can improve neurogenesis [68] and consequently can keep cognitive functions intact longer. All of this evidence confirms the importance of PE as a primary prevention tool.

Additionally, PE does not only work as a mechanism of prevention of neurodegenerative diseases, but its contribution even in patients with moderate and advanced AD is now known. Indeed, in 2007 Rolland et al. demonstrated some of the positive effects of PE in patients with moderate AD. Two years of regular activity in these patients determined improvements in walking time endurance, reduction of depression, incontinence minimization, increase in activities of daily living (ADL) and, more generally, improvements in all the symptoms typical of this disease [69]. Even more surprising are the results of a study conducted by Venturelli et al. on a group of patients over the age of 65 with a diagnosis of advanced AD who underwent, with the help of their caregivers, a 24-week walking program for at least 30 min per day. Through assessment scales such as the Barthel index, MMSE, the Performance Oriented Mobility Assessment (POMA) index, and constant oxygen saturation during walking (SpO_2_ > 85%), it was seen that exercise can slow down, even if for short periods of time, the progression of cognitive impairment and can improve performance of ADL [70]. 

So, PE could also now be considered a rehabilitative opportunity since it improves the abilities of AD patients.

Considering the absence of specific drugs to contrast AD, it seems to be very important to establish the dose–response effects of physical activity on cognition of these patients, and consequently, to find the combinations of frequency, intensity, time, and type of PE most useful to optimizing the results of this therapeutic intervention. Although this intent is obviously difficult to pursue, a recent meta-analysis [71] showed that, using MMSE as evaluation scale, moderate intensity of aerobic exercise seems to be the most effective intervention, if it is conducted at least one hour a week and for a duration range from 12 to 24 weeks. Moreover, the best cognitive improvement seems to be achieved with moderate intensity and frequency of physical activity: interventions conducted for up to 2 h had greater results than those conducted for more than 2 h per week; similarly, interventions conducted less than three times per week showed greater effect on improving cognition of AD patients compared to those conducted more than three times per week. Nevertheless, a threshold remains to be settled, since larger samples and longer follow-up are needed [72].

### 3.2. The Role of Physical Exercise in Parkinson’s Disease (PD)

Continuing the examination of the main neurodegenerative diseases that cause cognitive impairment, it is certainly necessary to pay attention to Parkinson’s disease as well. PD is a progressive neurodegenerative disease and is the second most common after AD, characterized by tremor, rigidity, bradykinesia, and postural instability. Its diagnosis is clinical even if a histopathological evaluation is necessary to identify α-synuclein contained in Lewy bodies or Lewy neurites [73]. PD etiology has not yet been clarified, but it seems to have a link to both genetic and environmental factors. The main distinctive morphological change in the PD brain is observed in the transverse sections of the brainstem, where almost all cases present with loss of the darkly pigmented area in the substantia nigra pars compacta (SNpc) and locus coeruleus. From a clinical point of view, alongside the aforementioned pathognomonic motor deficits, people with PD can exhibit different cognitive conditions, from normal cognition, through to early, mild subjective and objective decline, to mild, moderate and even severe PD dementia [74]. Severe dementia has a prevalence of 25–30% in PD patients and affects many cognitive functions, in particular, executive, attentional and visuospatial domains, but also memory [75]. 

In this context, PE seems to also be a primary prevention tool for PD. In a study based on a sample of over 200,000 participants, people who practiced high levels of physical activity from ages 15–39 years were less exposed to be diagnosed with PD later in life [76]. In another epidemiological study, Thacker et al. analyzed a cohort of about 143,000 individuals and found that people who practiced moderate to vigorous physical activity, such as bicycling, aerobics or tennis, had the lowest risk of PD during ten years of follow-up [77]. More specifically, PE seems to reduce the risk of developing cognitive impairment and PD, with a strong level of evidence for it being a protective factor for the latter [78]. 

However, PE is above all an opportunity for care in patients already suffering from PD. A comprehensive and recent review on the benefits of exercise training for PD patients highlighted the effects of different types of exercise for motor and cognitive dysfunctions: aerobic training, especially cycling, improves gait and cognitive function independently, but it also improves the motor learning ability, which translates into an improvement in motor functions applied to the gait [79].

Even more complex physical activities, such as dance, have proven effective not only in improving motor functions, but also in implementing executive functions, assessable with the Frontal Assessment Battery at bedside and Mental Rotation Task [80,81]. Additionally, Tai Chi, which is a composed of dance-like movements that are linked together in a complex sequence, provides benefits for psychological well-being and cognitive function [82]. Similarly, yoga, which is a sporting practice that includes postures and exercises of breathing and meditation, improves balance, mental and emotional health [83].

Moreover, PE is traditionally a pillar of rehabilitation treatments when it takes on the contours of therapeutic exercise. Therapeutic exercise, when organized in series with methodical exercises within programs aimed at specific rehabilitation objectives, has the ability to stimulate neuroplasticity at the level of the frontal lobe, counteracting cognitive impairment [84]. In particular, the exercises incorporating goal-based motor-skill learning improve motor-skill performance in PD through cognitive engagement.

The cognitive benefits of therapeutic exercise translate into general improvements in autonomy in PD patients. In fact, the overall functionality, measured with scales such as ADL [85] and Barthel Index [86], improves significantly; this attests to the actual ability of the exercise to rehabilitate these patients, that is, to give them back their skills in carrying out the fundamental activities of their daily life. The reduction of cognitive disabilities resulting from PD leads to an improvement in the quality of life [87].

A new frontier of neurorehabilitation deserves a special mention: virtual reality. Virtual reality plans to insert traditional exercise within virtual environments in which it is possible to exercise and monitor motor and cognitive functions in an easier and more precise way [88]. In PD patients, this type of sensorineural stimulation improves motor functions in terms of balance and gait, but above all increases executive functions [89]. In particular, virtual reality makes the training more complete, since it leads patients to simultaneously exercise multiple cognitive processes, linking them in sequences aimed at achieving specific goals, including those related to movement. More specifically, attention activation, information acquiring and processing, movement planning and sensory integration are contextually inserted into virtual contexts that require that they be applied to the daily activities of patients, in order to increase personal autonomy [90].

Additionally, PE can also limit the side effects such as wearing off and dyskinesia induced by anti-PD therapeutics, improving and prolonging the therapies’ effectiveness [91]. Moreover, physical activity reduces risk of other geriatric diseases such as diabetes, hypertension and cardiovascular disease, which may also contribute to PD pathogenesis [92]. In summary, PE is now considered to be a complementary strategy to PD medications. 

## 4. Conclusions

PE is an amazing health tool for the human brain, representing an opportunity for treatment and rehabilitation in patients suffering from cognitive impairment caused by neurodegenerative diseases.

As described in this article, there is already evidence of how PE acts positively at the neuroendocrine and biochemical level, and which beneficial clinical implications derive from it.

Nevertheless, it is desired that new studies investigate the functional mechanisms of physical exercise at the brain level, revealing the fascinating aspects that remain unknown but could allow us to implement its therapeutic potential.

## Data Availability

The datasets used and/or analyzed during the current study will be made available upon reasonable request to the corresponding author, L.C.
